# Mast Cells Mobilize Myeloid-Derived Suppressor Cells and Treg Cells in Tumor Microenvironment via IL-17 Pathway in Murine Hepatocarcinoma Model

**DOI:** 10.1371/journal.pone.0008922

**Published:** 2010-01-27

**Authors:** Zhuoshun Yang, Biao Zhang, Dapeng Li, Meng Lv, Chunmei Huang, Guan-Xin Shen, Bo Huang

**Affiliations:** 1 Department of Biochemistry & Molecular Biology, Tongji Medical College, Huazhong University of Science & Technology, Wuhan, The People's Republic of China; 2 Department of Immunology, Tongji Medical College, Huazhong University of Science & Technology, Wuhan, The People's Republic of China; Charité-Universitätsmedizin Berlin, Germany

## Abstract

Tumor immunosuppression is commonly braided with chronic inflammation during tumor development. However, the relationship between immunosuppression and inflammation in tumor microenvironment is still unclear. We have demonstrated that mast cells are accumulated and exacerbate the inflammation and immunosuppression in tumor microenvironment via SCF/c-kit signaling pathway. Here, we further elucidate the underlying mechanism, which involves both myeloid-derived suppressor cells (MDSCs) and regulatory T (Treg) cells. Our data showed that mast cells mobilized the infiltration of MDSCs to tumor and induced the production of IL-17 by MDSCs; MDSCs-derived IL-17 indirectly attracted Treg cells, enhanced their suppressor function, and induced the IL-9 production by Treg cells; in turn, IL-9 strengthened the survival and protumor effect of mast cells in tumor microenvironment. Our findings disclose a closed loop among mast cells, MDSCs and Treg cells in tumor microenvironment, which provides a new insight into the paralleled developments of inflammation and immunosuppression in tumor microenvironment. Based on these findings, we propose that targeting tumor inflammation might be a potential strategy to reverse the immunosuppression of tumor microenvironment, thus facilitating cancer immunotherapy.

## Introduction

A major challenge for cancer immunotherapy comes from the tumor-induced immunosuppression, which dampens cytotoxic activities of T lymphocytes and natural killer (NK) cells [1], [2]. Various immunosuppressive ways are exploited by tumors. However, why tumors have such versatile abilities to build an immunosuppressive microenvironment is still incompletely understood. During tumor development, tumor immunosuppression is commonly braided with “smoldering” inflammation [3], [4]. The latter probably is the driving force. Like normal tissues, tumors also need immune regulation to avoid the catastrophic damage from the uncontrolled inflammation. Therefore, in response to smoldering inflammation of tumors, multiple immunosuppressive cell types are mobilized to tumor. Among them, Treg cells and MDSCs are pivotal [5], [6].

Treg cells are distinct lymphocyte lineage endowed with regulatory properties in maintaining immunological tolerance. The expression of transcription factor Foxp3 is the most distinctive marker for Treg cells [7]. MDSCs are a heterogeneous population of immature myeloid cells originated from bone marrow [8], [9]. MDSCs in mouse are marked by Gr-1 and CD11b or more specifically by Gr-1 and CD115 (M-CSFR) [10]. Both Tregs and MDSCs may be directly involved in immune unresponsiveness via multiple mechanisms. The means by which Treg cells suppress tumor-specific T cells includes 1) secretion of suppressor cytokines IL-10 and TGF-β [11]; 2) suppression of the function of APC through CTLA4 pathway [12]; 3) hydrolysis of extracellular ATP to inhibitory adenosine by CD39 and CD73 [13]; and 4) transferring inhibitory cAMP from Treg cells to effector T cells through gap junction [14]. On the other hand, MDSCs are capable of inhibiting effector T cells by: 1) IFN-γ-dependent nitric oxide (NO) production [15]; 2) IL-4-dependent arginase 1 synthesis [16]; 3) inducing the loss of CD3ζ signaling [17]; 4) suppression of the T-cell response through reactive oxygen species [18]–[20]; and 5) mediating the development of Treg cells [10]. Regardless of such well defined immunosuppressive effects, it is unclear how Treg cells and MDSCs communicate with each other, and how tumor-infiltrating Treg cells and MDSCs are regulated in tumor microenvironment. In addition, MDSCs are reported to be related to inflammation [21]–[23]. However, whether MDSCs may directly contribute to tumor inflammation remains unknown.

Mast cells are critical innate immune cell type, which can also function as immune regulatory cells [24], [25]. We recently demonstrated that mast cells were accumulated in tumor microenvironment via SCF/c-kit signaling pathway, leading to the exacerbation of the inflammation and immunosuppression in tumor microenvironment [26]. In this study, we further investigated the mechanism by which mast cells mediate tumor inflammation and immunosuppression. We found that mast cells mobilized the infiltration of MDSCs to tumor and induced the production of IL-17 by MDSCs; MDSCs-derived IL-17 indirectly attracted Treg cells, enhanced their suppressor function, and induced the IL-9 production by Treg cells; in turn, IL-9 strengthened the survival and protumor effect of mast cells in tumor microenvironment. Thus, these findings indicate an intrinsic relationship among mast cells, MDSCs and Treg cells in tumor microenvironment.

## Results

### Regulation of Tumor-Infiltrating MDSCs by Mast Cells

**Figure 1:**
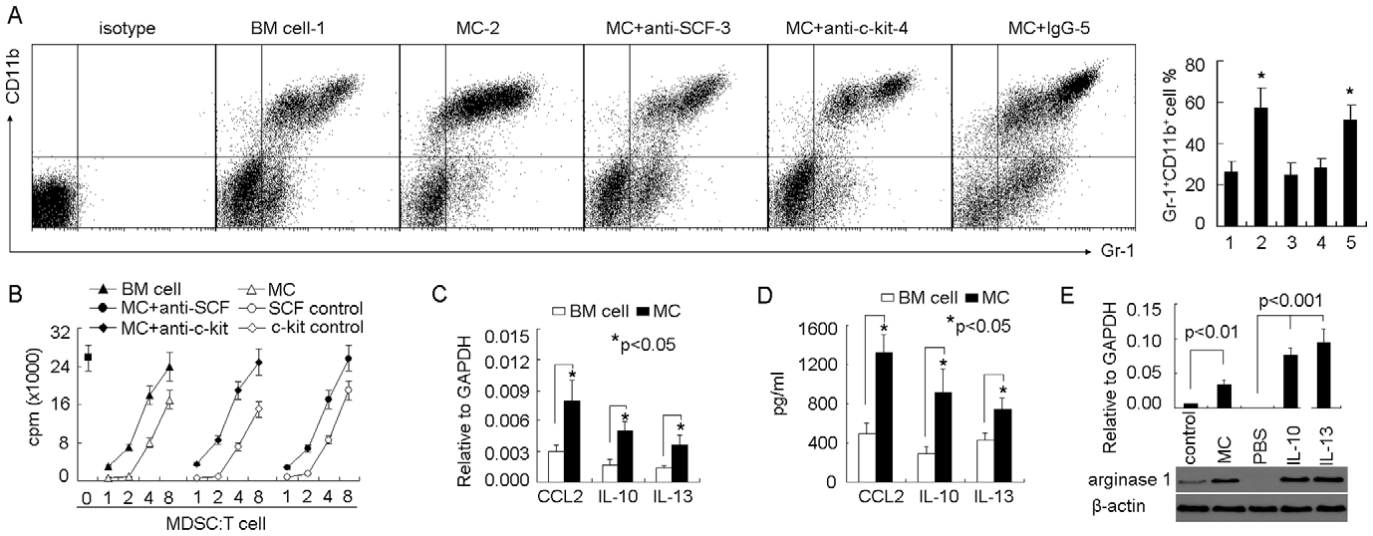
The regulation of tumor-infiltrating MDSCs by mast cells. (A) Mast cells promoted the infiltration of MDSCs into tumor microenvironment. 5×10^6^ BMMCs with or without anti-SCF or c-kit antibodies, were injected into tumor-bearing mice by i.v. injection. Bone marrow cells were used as control. Seven days later, the tumor-infiltrating lymphocytes were used to analyze Gr-1^+^CD11b^+^ MDSCs by flow cytometry. The left shown was the representative of FACS profiles. The right shown was the combined reproducible data (n = 6). *, *P*<0.05, compared to control. (B) Mast cells promoted the suppressive function of MDSCs in tumor microenvironment. BMMCs with or without antibodies, were injected into tumor-bearing mice (n = 6). Seven days later, tumor-infiltrating MDSCs were isolated as described in *Materials and Methods* and the suppression assay was performed as described in *Materials and Methods*. (C and D) Mast cells upregulated the expressions of CCL2, IL-10 and IL-13 in tumor microenvironment. BMMCs were injected into tumor-bearing mice. Seven days later, tumor tissues were used to analyze CCL2, IL-10 and IL-13 expressions by real time RT-PCR (C) and ELISA (D). (E) The regulation of arginase 1 by mast cells. Lane 1–2: tumor tissues from BMMC group or control were used to analyze arginase 1 expression by real time-RT-PCR and western blot. Lane 3–5: the cultured MDSCs were treated with PBS or IL-10 (20 ng/ml) or IL-13 (20 ng/ml) for 48 h, and then used for the analysis of arginase 1 expression.

We previously demonstrated that bone marrow-derived mast cells (BMMCs) effectively migrate to H22 hepatocarcinoma cell line-inoculated tumor site after tail vein injection [26]. Using this model, we initially examined the influence of mast cells on tumor-infiltrating MDSCs. BMMCs were injected into H22 tumor-bearing mice (5×5 mm). Seven days later, we analyzed the tumor-infiltrated Gr-1^+^CD11b^+^ MDSCs, and found that the proportion of MDSCs in tumor was significantly increased in BMMC group, compared to bone marrow cell control group (Fig. 1A). To exclude the contamination by MDSCs, we additionally labeled BMMCs with CFSE and injected cells to mice. The flow cytometric analysis indicated that the CFSE^+^ cells did not express MDSC's markers Gr-1 and CD11b (data not shown), suggesting no MDSC contamination during adoptive transfer of BMMCs. To determine the function of MDSCs, we isolated tumor MDSCs for suppression assay. We found that the injection of BMMCs enhanced the suppressor function of MDSCs on T cell proliferation (Fig. 1B). Our previous study showed that SCF/c-kit signaling is critical for the protumor effect of mast cells [26]. In line with this, we here also found that SCF neutralization or c-kit blockade impaired the effect of BMMCs on MDSCs (Fig. 1, A and B).

To explain the above effect of BMMCs on MDSCs, we selectively analyzed several factors relative to the migration and function of MDSCs, and found that the injection of BMMCs increased chemokine CCL2 level in tumor and upregulated arginase 1 expression in MDSCs (Fig. 1, C–E). In addition, the levels of IL-10 and IL-13 were also increased in tumor after BMMC injection (Fig. 1, C and D). Interestingly, the addition of either IL-10 or IL-13 upregulated arginase 1 expression in the *in vitro* cultured MDSCs (Fig. 1E), consistent with the previous report of upregulation of arginase 1 by Th2 cytokines [16]. CCL2 signaling has been reported to mediate the migration of MDSCs [27] and arginase 1 is an important pathway for mediating the suppressor function of MDSCs [28]. Therefore, mast cells probably promote the migration and suppressor function of tumor MDSCs by regulating the expressions of CCL2 and Th2 cytokines.

### Mast Cells Regulate MDSCs through IL-17 Pathway and Induce IL-17 Expression in MDSCs

**Figure 2:**
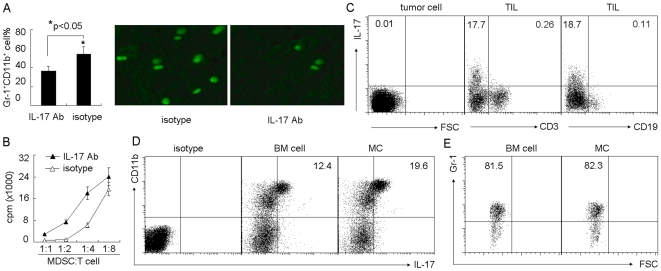
Mast cells regulate MDSCs through IL-17 pathway. (A) Blockade of IL-17 prevented mast cell-mediated MDSC infiltration to tumor. 5×10^6^ BMMCs were injected into tumor-bearing mice by i.v. injection. IL-17 neutralizing antibody was i.p. injected to the mice 1 h, 2 days and 5 days after BMMCs injection. On day 7, the tumor-infiltrating lymphocytes were used to analyze Gr-1^+^CD11b^+^ MDSCs by flow cytometry (left). In addition, IL-17 neutralizing antibody was i.p. injected to the mice 24 h and 1 h before BMMCs injection. 2×10^6^ CFSE-labeled MDSCs were injected into the mice two days later. The tumor tissues were surgically excised, and frozen sections were prepared and analyzed by fluorescence microscopy (right). (B) Blockade of IL-17 attenuated mast cell-mediated MDSC suppressive function. BMMCs were injected into tumor-bearing mice. IL-17 neutralizing antibody was i.p. injected to the mice at different time points. On day 7, tumor-infiltrating MDSCs were isolated for the suppression assay. (C) IL-17 was not expressed by H22 tumor cells, T cells or B cells. BMMCs were injected into tumor-bearing mice. Seven days later, tumor cells and TILs were isolated, respectively. The expression of IL-17 was analyzed by flow cytometry. (D and E) Mast cells upregulated the expression of IL-17 by MDSCs. Seven days after BMMCs injection, the isolated TILs were used for IL-17 expression analysis. The data showed the upregulation of IL-17 by CD11b^+^ cells in BMMC group (D), and most of the gated IL-17^+^ cells expressed Gr-1 marker (E).

Considering that the inflammation is capable of regulating CCL2 and Th2 cytokines and mast cells are the key inflammatory cells, we speculated that inflammation pathway was involved in mast cells-mediated regulation of MDSCs. Interestingly, IL-17, a critical inflammatory cytokine [29], was reported to be upregulated by mast cells in tumor microenvironment [26]. In this context, we wondered whether mast cells regulated MDSCs through IL-17 pathway. To test this, we injected BMMCs into tumor-bearing mice and the IL-17 neutralizing antibody was administrated at different times after BMMCs injection. We found that the blockade of IL-17 with the antibody prevented BMMCs-mediated MDSC infiltration to tumor and decreased the suppressive activity of MDSCs (Fig. 2, A and B), suggesting that mast cells regulate MDSCs through IL-17 pathway. We then investigated the cellular source of IL-17 induced by BMMCs. We isolated tumor cells from tumor tissue for the intracellular staining. IL-17^+^ tumor cells were not detectable (Fig. 2C). Although Th17 cells are considered as the main cell type for IL-17 production, in our tumor model, we found that few CD3^+^ T cells expressed IL-17 (Fig. 2C). Meanwhile, IL-17 was not expressed by CD19^+^ cells (Fig. 2C). Therefore, IL-17 might be expressed by innate immune cells. We have reported IL-17 expression by CD11b^+^ cells in inflammatory allergic lung tissue [30]. In line with this, we found that BMMCs augmented CD11b^+^ cells expressing IL-17 in tumor microenvironment (Fig. 2D). Further analysis confirmed that most IL-17^+^CD11b^+^ cells were Gr-1 positive, and Gr-1^+^CD11b^+^ MDSCs were the main cellular source of IL-17 in tumor microenvironment (Fig. 2E). Thus, these data disclosed an unknown role of MDSCs in that MDSCs migrate to tumor site and participate in tumor inflammation by producing IL-17 in response to the remodeled tumor inflammation by mast cells.

### Regulation of Treg Cells by Mast Cells in Tumor Microenvironment

**Figure 3:**
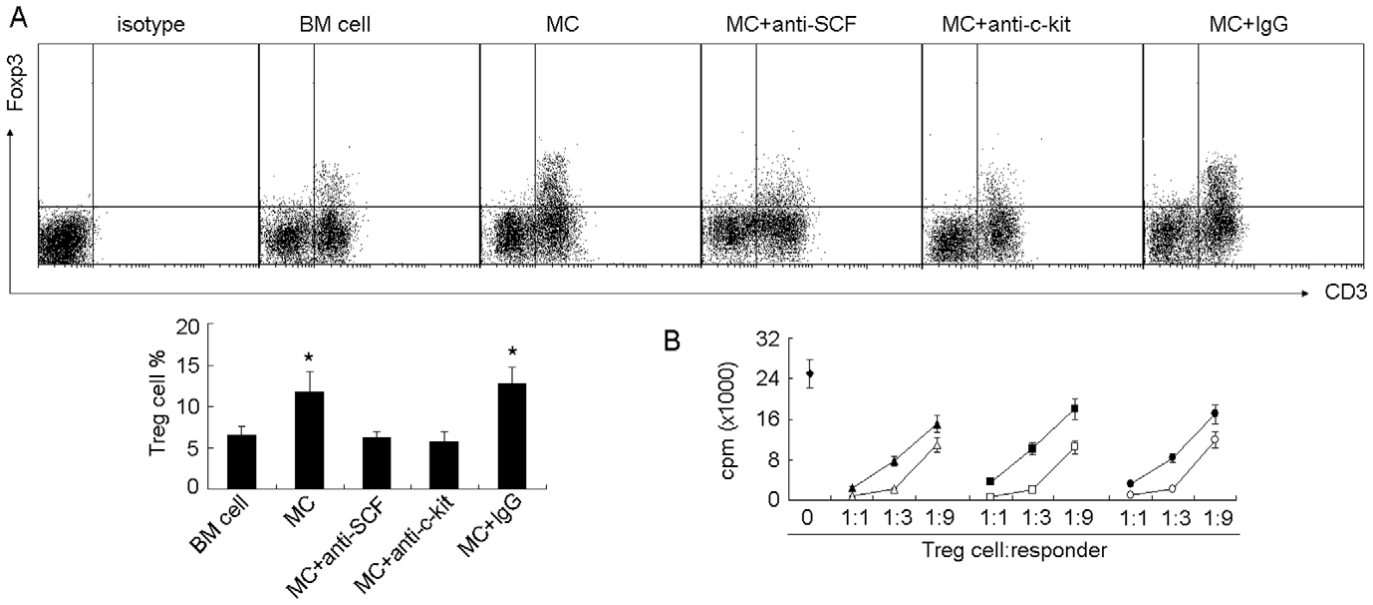
Mast cells regulate the infiltration and function of Treg cells in tumor microenvironment. (A) Mast cells regulate the infiltration of Treg cells. 5×10^6^ BMMCs with or without anti-SCF or c-kit antibodies, were injected into tumor-bearing mice by i.v. injection. Bone marrow cells were used as control. Seven days later, the tumor-infiltrating lymphocytes were used to analyze CD3^+^Foxp3^+^ Treg cells by flow cytometry. The left shown was the representative of FACS profiles. The right shown was the combined reproducible data (n = 6), *, *P*<0.05, compared to BM cell control. (B) Mast cells regulate the suppressive function of Treg cells. BMMCs with or without antibodies, were injected into tumor-bearing mice (n = 6). Seven days later, tumor-infiltrating Treg cells were isolated as described in *Materials and Methods* and the suppression assay was performed as described in *Materials and Methods*.

Next, we investigated the influence of mast cells on tumor-infiltrating Treg cells. Seven days after the i.v. injection of BMMCs, the frequency of Treg cells in tumor microenvironment was significantly increased (12.2% versus 18.7%, Fig. 3A); and the suppression of Treg cells on T cell proliferation was also enhanced (Fig. 3B). Consistently, the prevention of BMMC migration to tumor by either SCF neutralization or c-kit blockade resulted in the no effect of BMMCs on Treg cells (Fig. 3, A and B). Therefore, these data suggested that mast cells regulate the infiltration and suppressor function of Treg cells in tumor microenvironment.

### Mast Cell-Induced IL-17 Mediates Treg Cell Infiltration via Upregulating Chemokines CCL17 and CCL22

**Figure 4:**
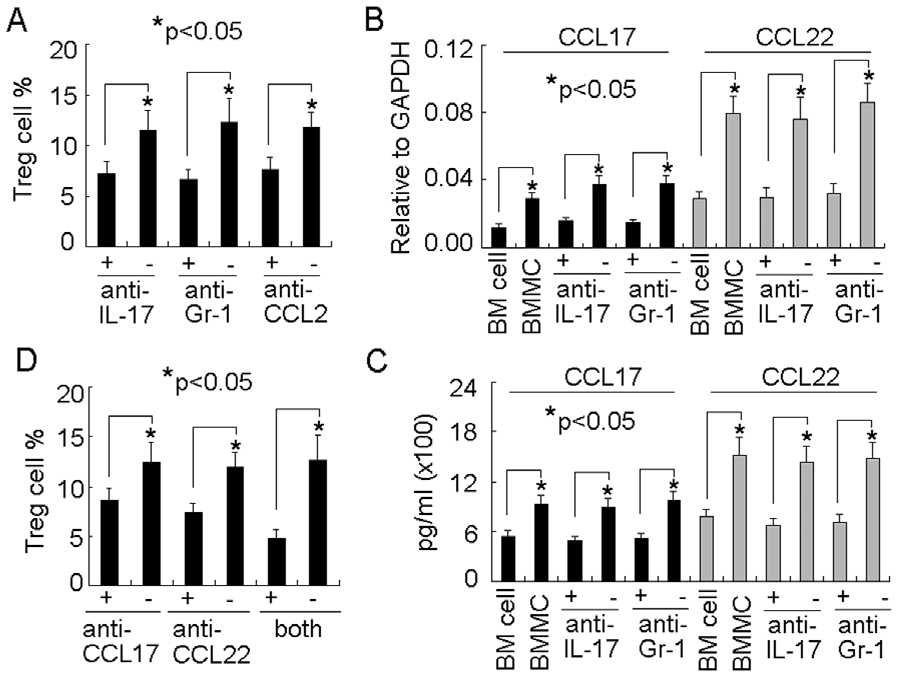
Mast cell-induced IL-17 mediates Treg cell infiltration via upregulating chemokines CCL17 and CCL22. (A) The interference of IL-17 impaired the effect of mast cells on Treg cell infiltration. 5×10^6^ BMMCs were injected into tumor-bearing mice by i.v. injection. IL-17 or CCL2 neutralizing antibody or Gr-1 depleting antibody was i.p. injected to the mice 1 h, 2 days and 5 days after BMMCs injection. On day 7, the tumor-infiltrating lymphocytes were isolated to analyze CD3^+^Foxp3^+^ Treg cells by flow cytometry. The results were combined from three mice. (B and C) Mast cells-induced IL-17 upregulated CCL17 and CCL22 expressions in tumor microenvironment. BMMCs were injected into tumor-bearing mice. IL-17 or Gr-1 antibody was i.p. injected to the mice at different time points. Seven days after BMMCs injection, the tumor tissues were used to analyze CCL17 and CCL22 expressions by real time RT-PCR (B) and ELISA (C). (D) The effect of CCL17 and CCL22 on Treg cell infiltration. BMMCs were injected into tumor-bearing mice. CCL17 or CCL22 neutralizing antibody was i.p. injected to the mice 1 h, 2 days and 5 days after BMMCs injection. On day 7, the tumor-infiltrating lymphocytes were isolated to analyze CD3^+^Foxp3^+^ Treg cells by flow cytometry. The results were combined from three mice.

Migration of Treg cells to inflammation site might be to avoid the catastrophic damage from the uncontrolled inflammation by suppressing immune responses. In this regard, we speculated that mast cell-exacerbated tumor inflammation facilitated Treg cell infiltration. To test this, we here concentrated on IL-17. The neutralization of IL-17 resulted in the no effect of BMMCs on Treg cell infiltration (Fig. 4A), Since MDSCs was the cellular source of IL-17 in this tumor model, we therefore depleted MDSCs by i.p. injection of anti-Gr-1 depleting antibody [31] or i.p. injected anti-CCL2 neutralizing antibody to inhibit MDSC migration to tumor [27]. Consistently, we found that either MDSC depletion or migration blockade blunted the effect of BMMCs on Treg cell infiltration (Fig. 4A). These data suggested that BMMCs exert its effect on Treg cells through IL-17 pathway.

Next, we investigated the underlying mechanism by which BMMCs-induced IL-17 facilitated Treg cell infiltration. We paid the attention on chemokines CCL17 and CCL22, the selective chemoattractants for Treg cells [32]–[34]. We found that BMMC injection increased the expression of CCL17 and CCL22 in tumor microenvironment, evaluated by real time RT-PCR (Fig. 4B), which could be blunted by IL-17 neutralization or blocking MDSC migration (Fig. 4B). A consistent result was observed by analyzing the protein levels of CCL17 and CCL22 by ELISA (Fig. 4C). Furthermore, the neutralization of CCL17 or CCL22 could prevent Treg cell infiltration to tumor (Fig. 4D). Therefore, mast cells-induced IL-17 increased the level of CCL17 and CCL22 in tumor microenvironment, which then chemoattracted Treg cells to tumor.

### Mast Cell-Induced IL-17 Enhances Suppressor Function of Treg Cells via Upregulating CD39 and CD73

**Figure 5:**
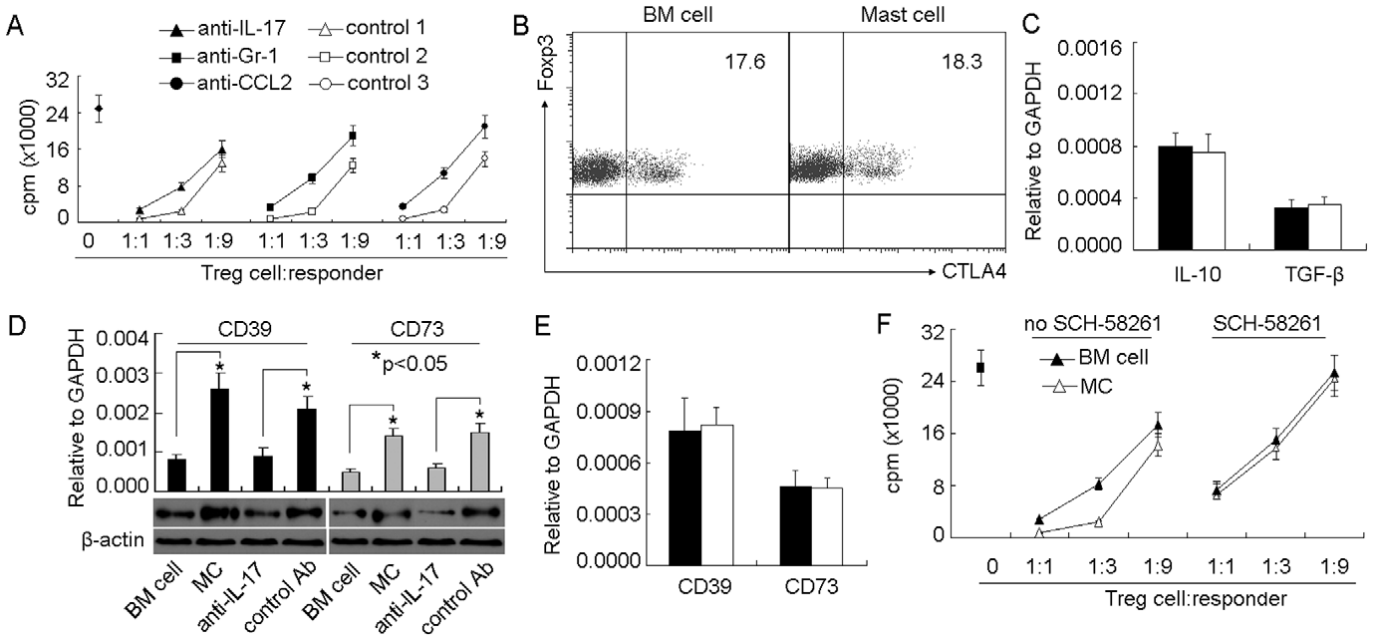
Mast cell-induced IL-17 enhances suppressor function of Treg cells via upregulating CD39 and CD73. (A) The interference of IL-17 impaired the effect of mast cells on the suppressive function of Treg cells. 5×10^6^ BMMCs were injected into tumor-bearing mice by i.v. injection. IL-17 or CCL2 neutralizing antibody or Gr-1 depleting antibody was i.p. injected to the mice 1 h, 2 days and 5 days after BMMCs injection. On day 7, tumor-infiltrating Treg cells were isolated for suppression assay. (B and C) Mast cells had no effect on Treg cells expressing CTLA-4, IL-10 or TGF-β. BMMCs were injected into tumor-bearing mice. Seven days later, the tumor-infiltrating lymphocytes were isolated for the analysis of CTLA-4 by flow cytometry. The data showed the gated CD3^+^Foxp3^+^ cells. (B) or IL-10 and TGF-β by real time RT-PCR (C). (D) Mast cells upregulated the expressions of CD39 and CD73 by Treg cells. BMMCs were injected into tumor-bearing mice with IL-17 antibody or control antibody. Seven days later, the tumor-infiltrating Treg cells were isolated for the analysis of CD39 and CD73 by real time RT-PCR and western blot. (E) IL-17 had no direct effect on Treg cells. IL-17 (20 ng/ml) was added to the cultured Treg cells for 12 hours. The cells were collected for the analysis of CD39 and CD73 by real time RT-PCR. *F*, Blockade of adenosine signaling pathway impaired mast cell-enhanced Treg cell function. BMMCs were injected into tumor-bearing mice. BM cells were used as control. Seven days later, the tumor-infiltrating Treg cells were isolated for suppression assay in the presence or absence of adenosine receptor A_2A_ antagonist SCH-58261 (100 ng/ml). The data shown were the representative of 2 independent experiments in which the similar results were obtained.

Besides its effect on Treg cell infiltration, we also found that IL-17 affected the suppressive function of Treg cells in tumor microenvironment. IL-17 neutralization effectively blunted the effect of BMMCs on Treg cell function (Fig. 5A). A consistent result was observed, if depleting MDSCs or blocking MDSC migration (Fig. 5A). Therefore, MDSC-derived IL-17 enhanced the suppressive function of Treg cells. Here, we further studied the molecular basis. We wondered whether inhibitory molecule CTLA4 expressed by Treg cells was the reason. However, the flow cytometric analysis did not show the different expression of CTLA4 between BMMC group and control (Fig. 5B). In addition, the enhanced suppressive function seemed not be explained by inhibitory cytokines IL-10 and TGF-β, since their mRNA levels were not altered in Treg cells evaluated by real time RT-PCR analysis (Fig. 5C). Recently, ectoenzymes CD39 and CD73 were demonstrated to play important role in Treg cell suppressive function through hydrolyzing nucleotide to adenosine, leading to immune inhibitory signal transduction via adenosine receptor A_2A_ [13], [35]. Here, we found that BMMC injection upregulated the expressions of CD39 and CD73 in Treg cells and IL-17 neutralization impaired this effect (Fig. 5D). Interestingly, in vitro study showed that IL-17 had no effect on Treg cells expressing CD39 and CD73 (Fig. 5E), suggesting an indirect effect of IL-17 on Treg cells *in vivo*. To address whether the increased CD39 and CD73 enhanced the suppressive function of Treg cells, we used adenosine receptor A_2A_ antagonist SCH-58261 for the *in vitro* suppression assay, since adenosine is the hydrolytic product of CD39 and CD73 and mediates the immunosuppression [13]. As expected, blocking adenosine pathway attenuated the suppressive function of Treg cells enhanced by BMMC injection (Fig. 5F). Taken together, these data suggested that IL-17 induced by mast cells leads to Treg cells upregulating CD39 and CD73 expression for an enhanced suppressive function in tumor microenvironment.

### The Production of IL-9 by Treg Cells Is Required for Mast Cell-Mediated Protumor Effect in Tumor Microenvironment

**Figure 6:**
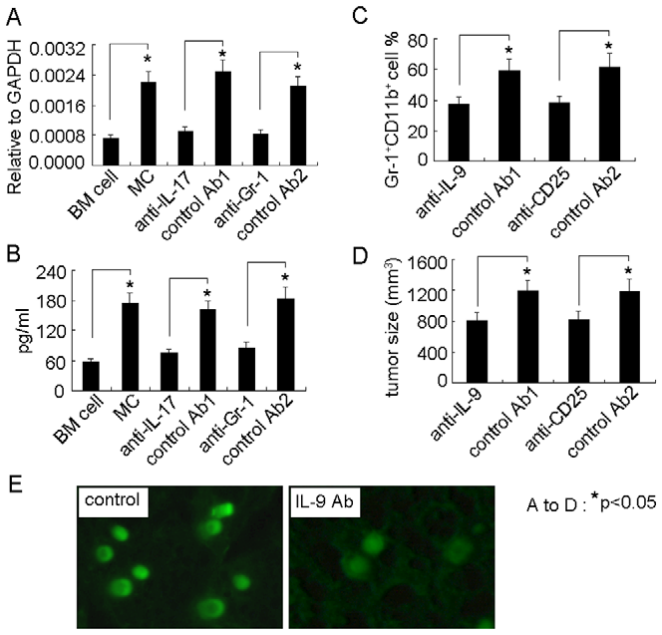
IL-9 strengthens the survival and protumor effect of mast cells in tumor microenvironment. (A and B) Mast cells upregulated the expressions of IL-9 by Treg cells. BMMCs were injected into tumor-bearing mice with IL-17 antibody or control antibody. Seven days later, the tumor-infiltrating Treg cells were isolated for the analysis of IL-9 by real time RT-PCR (A). Or the isolated Treg cells were cultured for 48 hours. The supernatant was used for IL-9 ELISA assay (B). (C and D) The interference of IL-9 signaling affected mast cell-mediated MDSC infiltration and mast cell-promoted tumor growth. 5×10^6^ BMMCs were injected into tumor-bearing mice (n = 6) by i.v. injection. IL-9 neutralizing antibody or CD25 depleting antibody was i.p. injected to the mice 1 h, 2 days and 5 days after BMMCs injection. On day 12, the tumor-infiltrating lymphocytes were used to analyze Gr-1^+^CD11b^+^ MDSCs by flow cytometry (C), and the tumor growth was monitored by measuring the length (L) and width (W) of tumors. The volume (V) of the tumor was calculated by the formula V = (L×W2)/2 (D). (E) IL-9 affected the survival of mast cells in tumor microenvironment. 1×10^6^ CFSE-labeled BMMCs were directly injected into tumor tissue with multiple injection sites. IL-9 neutralizing antibody was i.p. injected to the mice 1 h, 2 days and 5 days after BMMCs injection. The tumor tissues were surgically excised on day 7 for fluorescent analysis of frozen sections.

Given that Treg cells produce IL-9 for immune suppression through the activation of mast cells in an allograft model [36], and that IL-9 plays an important role in mast cell survival, we here further speculated that the involvement of IL-9 by Treg cells in mast cell-mediated tumor promotion. Three days after BMMC injection, the upregulation of IL-9 in Treg cells was observed, evaluated by real time RT-PCR and ELISA analysis (Fig. 6, A and B). However, the neutralization of IL-17 or depletion of MDSCs blunted such upregulation (Fig. 6, A and B), suggesting that mast cells enhance Treg cells expressing IL-9 via IL-17 signaling pathway in tumor microenvironment. Next, we examined the influence of IL-9 on mast cells. The interference of IL-9 signaling by either IL-9 neutralizing antibody or depleting Treg cells weakened BMMCs-mediated MDSC infiltration toward tumor (Fig. 6C), and attenuated BMMCs-enhanced tumor growth (Fig. 6D). Moreover, the neutralization of IL-9 decreased the survival time of BMMCs in tumor microenvironment (Fig. 6E). Taken together, these data suggested that cytokine IL-9 by Treg cells plays an important role in mast cell-mediated tumor promotion in tumor microenvironment.

## Discussion

In our previous study, we demonstrated that mast cells infiltrating into tumor exerts a protumor effect by exacerbating the inflammation and immunosuppression in tumor microenvironment [26]. Here, we further elucidate the underlying mechanism, which involves two critical regulatory cell types: Treg cells and MDSCs.

The initiation and remodeling of tumor microenvironment still remains unclear. Our unpublished data indicated that mast cells may be recruited to tumor cell-inoculated site as early as two hours after inoculation, which further emphasized that mast cells are a key regulator for tumor microenvironment. To realize this, the recruitment of MDSCs by mast cells via CCL2/CCR2 axis probably is a crucial step. Mast cells have the potential to secrete diverse biologically active products upon exposure to a variety of immunological or nonimmunological stimuli [24], [25]. Here, we find that the activated mast cells remodel tumor inflammatory microenvironment by upregulating CCL2, IL-10 and IL-13, which are associated with the migration and function of MDSCs, respectively. Recently, chemokines SDF-1 and CXCL5 have been shown to play a role in MDSC migration to tumor microenvironment [37]. In our tumor model, however, BMMC injection did not alter the expressions of SDF-1 and CXCL5 in tumor microenvironment (data not shown). We previously reported that CCL2 is generally expressed in various tumor types of both human and mouse and CCR2 is expressed by MDSCs [27]. Movahedi et al. also reported that CCR2 is a marker for MDSCs [38]. On the basis of these findings, we propose that CCL2/CCR2 axis may be an important signaling pathway for MDSC recruitment to tumor microenvironment.

Besides effecting on tumor immune evasion, tumor-infiltrating MDSCs may also employ other ways for tumor promotion. Yang et al reported that MDSCs produce MMP9 for tumor angiogenesis [39]. In a breast cancer model, tumor-infiltrating MDSCs are shown to favor tumor cell invasion and metastasis (37). In the present study, we identify a new role of MDSCs in tumor microenvironment by producing proinflammatory cytokine IL-17. As a critical proinflammatory cytokine, IL-17 has attracted great attention recently. IL-17 acts on a broad range of cells to induce the expression of cytokines (IL-6, IL-8, GM-CSF, G-CSF), chemokines, and metalloproteinases [40]–[43]. It also cooperates with TLR ligands, IL-1β, and TNF-α to enhance inflammatory reactions and stimulate the production of β-defensins and other antimicrobial peptides [44]. Although we here did not elucidate the molecular basis of the upregulation of IL-17 in MDSCs by mast cells, we illuminated the influence of IL-17 on MDSCs and Treg cells. And these findings may lead to a better understanding on the relationship between inflammation and immune regulation. IL-17 inducted by tumor-infiltrating mast cells may profoundly mold the inflammatory microenvironment. To avoid the inflammation-induced tissue damage, regulatory cells such as MDSCs and Treg cells are recruited to tumor microenvironment by IL-17-induced upregulation of chemokines CCL2, CCL17 and CCL22. Furthermore, the suppressor functions of MDSCs and Treg cells are enhanced by IL-17-upregulated IL-10 and IL-13 in tumor microenvironment and CD39 and CD73 on Treg cells. Currently, the role of IL-17 in tumor is controversial [45]–[50]. According to our findings, we suggest a protumor role of IL-17 by remodeling tumor microenvironment. In addition, our findings also suggest that the inflammation and immunosuppression can be developed in a parallel manner.

Regardless of the extensive study on Treg cells and MDSCs in tumor immunology, the general question of how Treg cells and MDSCs interact in tumor microenvironment still remains unclear. The previous report indicated that MDSCs mediate the development of Treg cells through IL-10 and IFN-γ pathways [10]. Our present study further sheds light on this issue. In tumor microenvironment, MDSCs indirectly influence the migration and activity of Treg cells by producing IL-17. On the other hand, Treg cells producing IL-9 indirect influence MDSCs through IL-9-affected mast cell pathway. Therefore, MDSCs and Treg cells may interact through indirect ways in tumor microenvironment. However, the direct interaction between MDSCs and Treg cells needs further study.

**Figure 7:**
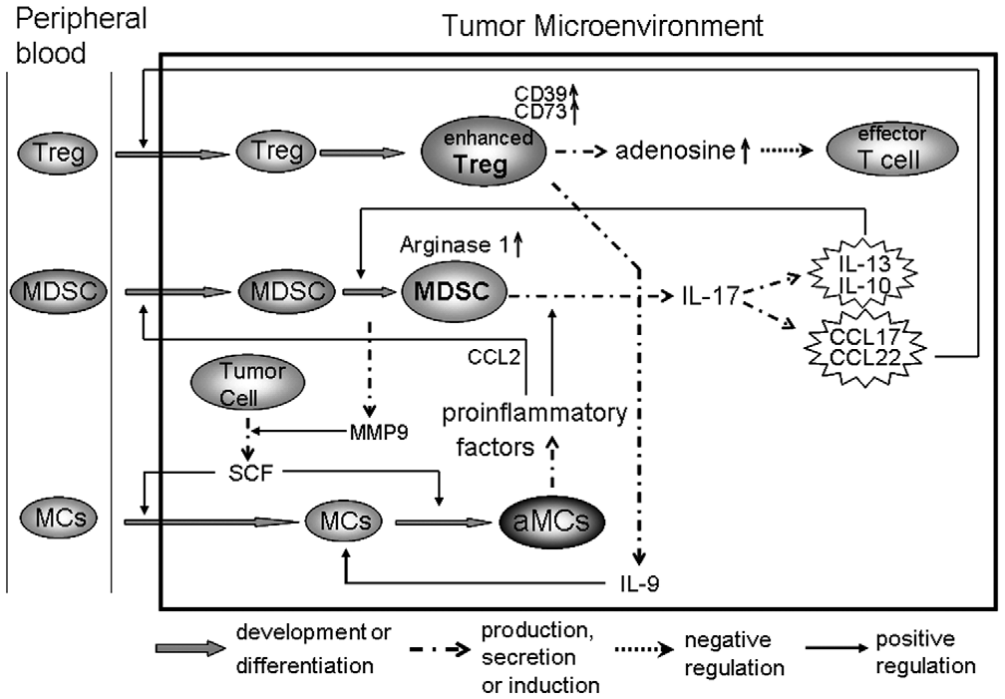
A model of the closed loop among mast cells, MDSCs and Treg cells in tumor microenvironment. Under the guidance of SCF/c-kit signaling, mast cells migrate to and are activated in tumor microenvironment; the activated mast cells release a panel of factors, leading to CCL2 production and IL-17 upregulation in MDSCs; CCL2 signaling recruits more MDSCs, leading to more IL-17 production; IL-17 strengthens tumor inflammatory microenvironment, leading to the upregulation of IL-9, IL-10, IL-13, CCL17, CCL22, CD39 and CD73; IL-10 and IL-13 induce arginase 1 expression by MDSCs; CCL17 and CCL22 attract the migration of Treg cells; CD39 and CD73 enhance suppressor function of Treg cells; IL-9 produced by Treg cells maintains the survival of mast cells; MDSCs release active MMP9, through which soluble SCF is generated, thus further facilitating the migration and activation of mast cells.

Based on our present and other findings [26], [39], we propose a closed loop among mast cells, MDSCs and Treg cells in tumor microenvironment (Fig 7), which provides a new insight into the relationship between inflammation and immunosuppression in tumor microenvironment. Our study also implies that targeting tumor inflammation might be a potential strategy to reverse the immunosuppression of tumor microenvironment, thus facilitating cancer immunotherapy.

## Materials and Methods

### Ethics Statement

All animal work was conducted according to relevant national and international guidelines. For details please refer to subsection entitled **Animals and cell lines**.

### Animals and Cell Lines

BALB/c mice, 6 to 8-week-old, were purchased from Center of Medical Experimental Animals of Hubei Province (Wuhan, China) for studies approved by the Animal Care and Use Committee of Tongji Medical College. Mouse hepatocarcinoma tumor cell line H22 was purchased from the China Center for Type Culture Collection (CCTCC, Wuhan, China), and cultured according to the guideline.

### Generation of Bone Marrow-Derived Mast Cells

Bone marrow cells were harvested from femurs of mice and cultured in RPMI 1640 supplemented with 10% FBS, 2 mM L-glutamine, 1 mM sodium pyruvate, 1 mM HEPES, 50 µM 2-ME, 100 U/ml penicillin, and 100 µg/ml streptomycin. The cells were cultured in the presence of IL-3 and SCF (10 ng/ml each, PeproTech, Rocky Hill, NJ), and the nonadherent cells were passaged every 3 days. 4 weeks later, the purity of mast cells was assessed by toluidine blue staining of cytocentrifuge preparations. Mast cells were identified by their morphological features and the presence of metachromatic granules. Only those preparations containing >98% mast cells were used in our studies and referred to as bone marrow-derived mast cells (BMMCs).

### Tumor Model

BALB/c mice were inoculated with H22 tumor cells by subcutaneous injection of 2×10^5^ cells to the left flank. 12 days later, the mice (n = 6 per group) with tumor size of ∼5×5 mm^2^ received 5×10^6^ BMMCs by i.v. injection. When indicated, the mice received i.p. injection of 100 µg of goat-anti-mouse SCF neutralizing antibody (IgG, R&D Systems, Minneapolis, MN) or goat IgG isotype control 24 h and 1 h before BMMCs injection, or received the i.v. injection of BMMCs mixed with 50 µg of rat anti-mouse c-Kit blocking antibody (eBioscience, San Diego, CA) or rat IgG2b isotype control.

### In Vivo Depletion Assay

Treg cells were depleted in vivo by i.p. injection of 100 µg of anti-mouse CD25 antibody (PC61.5; eBioscience). MDSCs were depleted in vivo by i.p. injection of 50 µg of anti-mouse Gr-1 antibody (RB6-8C5; eBioscience). To prevent the migration of MDSCs, the mice received i.p. injection of 100 µg of goat-anti-mouse CCL2 neutralizing antibody (IgG, R&D Systems, Minneapolis, MN).

### Isolation of Tumor Cells, Treg Cells and MDSCs

Tumors were digested with collagenase and hyaluronidase for 1 h at 37°C. After grinding with semifrosted slides and lysis of RBC, the dissociated cells were incubated on ice for 20 min, and then spun down at 500 rpm for 1 min. The cell pellet was washed and used as tumor cells. The suspension cells were underlaid with 5 ml of Lymphocyte-M solution, centrifuged (2,200 rpm for 20 minutes). Tumor-infiltrating lymphocytes (TILs) were harvested from the interface. Treg cells were isolated with Treg cell isolation kit (Miltenyi Biotec, Auburn, CA). The Gr-1^+^CD11b^+^ cells were sorted as MDSCs under stringent gating conditions (97–98% purity, BD FACSAriaTM cell sorter).

### Analysis of MDSCs and Mast Cells in Tumor Tissues

BALB/c mice were inoculated with H22 tumor cells as above. 12 days later, 5×10^6^ BMMCs were injected into tumor-bearing mice via tail vein, When indicated, the mice received i.p. injection of 100 µg of goat-anti-mouse IL-17 neutralizing antibody (R&D Systems, Minneapolis, MN) or isotype control 24 h and 1 h before BMMCs injection. 2×10^6^ CFSE-labeled MDSCs isolated from tumor-bearing mice bone marrow and spleen were injected into the above mice two days after BMMCs injection. The tumor tissues were surgically excised from mice 24 hours after the injection, and frozen sections were prepared and analyzed by fluorescence microscopy (200×, Leica DMI6000B, Wetzlar, Germany), using HC Plans objective lens and a Leica DFC300 FX camera. Image acquisition and processing were performed using Leica Application Suite software, version 2.3.4.R2.

As for mast cell analysis, 1×10^6^ CFSE-labeled BMMCs were directly injected into tumor tissue with multiple injection sites. The mice received i.p. injection of 100 µg of goat-anti-mouse IL-9 neutralizing antibody (R&D Systems) or isotype control 24 h before and 2 days and 5 days after BMMCs injection. The tumor tissues were surgically excised at different time points for fluorescent analysis of frozen sections.

### Analysis of Gene Expression by Real-Time RT-PCR

Total RNA was extracted from cells with TRIzol reagent (Invitrogen, Carlsbad, CA) or from tissues homogenized in TRIzol according to the manufacturer's instructions. For real time RT-PCR assays, the primers were designed with the Oligo Primer Analysis 4.0 software and the sequences were blasted (http://www.ncbi.nlm.nih.gov/BLAST/). 100 ng of total RNA was used for reverse transcription using Superscript II RNase H reverse transcriptase (Invitrogen) in a volume of 25 µl. Then 2 µl of cDNA was amplified with SYBR Green Universal PCR Mastermix (Bio-Rad, Richmond, CA) in duplicate. For sample analysis, the threshold was set based on the exponential phase of products, and CT value for samples was determined. The resulting data were analyzed with the comparative CT method for relative gene expression quantification against house keeping gene GAPDH.

### Western Blot Analysis

Cell lysates or tumor tissue homogenates (30 µg of total protein) and prestained molecular weight markers were separated by SDS-PAGE followed by transfer onto nitrocellulose membranes. The membranes were blocked in TBST (Tris-buffered saline with 0.5% of Triton X-100) containing 5% nonfat milk, and probed with primary antibodies. After incubation with the secondary antibody conjugated with horseradish peroxidase, membranes were extensively washed, and the immunoreactivity was visualized by enhanced chemiluminescence according to the manufacturer's protocol (ECL kit, Santa Cruz, Santa Cruz, CA). All antibodies were purchased from Santa Cruz (Santa Cruz, CA).

### Enzyme-Linked Immunoadsorbent Assay (ELISA)

For the assays of IL-10, IL-13, CCL2, CCL17, and CCL22, tumor tissues were homogenized in PBS (0.5 ml) containing 100 µm PMSF (Sigma), 1% (vol/vol) aprotinin (Sigma), 2 µg/ml leupeptin (Sigma), and 1 µg/ml pepstatin (Sigma). After centrifugation, the supernatant was assessed by ELISA kits (R & D Systems). For, IL-9 ELISA assay, the isolated Treg cells from tumor tissue were cultured in 96-plate well. 24 hr later, the supernatant was assessed by ELISA kits (R & D Systems).

### Flow Cytometric Analysis

The isolated tumor-infiltrating lymphocytes were stained with FITC-conjugated rat-anti-mouse Gr-1 and PE-Cy7-conjugated rat-anti-mouse CD11b for the analysis of MDSCs, or with PE-Cy7-conjugated hamster-anti-mouse CD3 and FITC-conjugated rat-anti-mouse Foxp3 for the analysis of Treg cells. All fluorophore-conjugated Abs and the corresponding isotypes were purchased from eBioscience. The stained cells were used for flow cytometric analysis (BD LSR II).

For intracellular staining, after cellular surface staining cells were fixed and permeabilized with Fix/Perm solution (eBioscience). The cells were then resuspended in Perm buffer and incubated with APC-labeled anti-mouse IL-17 antibody or FITC-conjugated rat-anti-mouse Foxp3 (eBioscience) at room temperature in the dark for 20 min.

### MDSC Suppression Assay

To assess the suppressive activity of MDSCs, splenic T cells were isolated from spleen of naïve mice with T cell-enrichment column (R & D systems). The isolated T cells (2×10^4^) were cultured with irradiated MDSCs at different ratios in the presence of irradiated splenocytes (5×10^4^) and anti-CD3 antibody (1 µg/ml) in 96-well microplates. [^3^H]thymidine was added during the last 10 hours of 72-hour culture for the determination of T cell proliferation.

### Treg Cell Suppression Assay

Naïve splenic T cells were used as responder cells. A total of 2×10^4^ responder cells were co-cultured with isolated Treg cells at different ratio for 3 days in the presence of 5×10^4^ irradiated APCs (splenocytes) and anti-CD3 (1 µg/ml). [^3^H]thymidine was added during the last 10 hours of 72-hour culture.

### Statistics

Results were expressed as mean value ± SD and interpreted by ANOVA-repeated measure test. Differences were considered to be statistically significant when *P*<0.05.
